# Effects of Curcumin on Radiation/Chemotherapy-Induced Oral Mucositis: Combined Meta-Analysis, Network Pharmacology, Molecular Docking, and Molecular Dynamics Simulation

**DOI:** 10.3390/cimb46090625

**Published:** 2024-09-20

**Authors:** Zhi-Xing Chen, Ya-Shi Qin, Bang-Hui Shi, Bi-Yun Gao, Ren-Chuan Tao, Xiang-Zhi Yong

**Affiliations:** 1 College of Stomatology, Guangxi Medical University, Nanning 530021, China; 15296543810@163.com (Z.-X.C.); 18881737072@163.com (Y.-S.Q.); 18350072218@163.com (B.-H.S.); joely918@163.com (B.-Y.G.); 2 Guangxi Health Commission Key Laboratory of Prevention and Treatment for Oral Infectious Diseases, Nanning 530021, China

**Keywords:** curcumin, radiation/chemotherapy-induced oral mucositis, meta-analysis, network pharmacology, molecular docking, molecular dynamics simulation

## Abstract

The study aims to investigate the effects of curcumin on radiation/chemotherapy-induced oral mucositis (R/CIOM) and preliminarily explore its mechanism. Randomized controlled trials were identified from the PubMed, Embase, Web of Science, Cochrane Library, Medline, and Google Scholar databases. RevMan 5.4 was used for statistical analysis to calculate the combined risk ratios (RRs). The mechanism was analyzed through network pharmacology, molecular docking, and a molecular dynamics simulation. The targets of curcumin were collected in HERB, PharmMapper, Targetnet, Swiss Target Prediction, and SuperPred. OMIM, GeneCards, and Disgenet were used to collect relevant targets for R/CIOM. Cytoscape software 3.8.0 was used to construct the component-target-pathway network. Protein–Protein Interaction (PPI) networks were constructed using the STRING database. GO and KEGG enrichment analyses were performed by Metascape. AutoDock Vina 4.2 software was used for molecular docking. The molecular dynamics simulation was performed by Gromacs v2022.03. It is found that 12 studies involving 565 patients were included. Meta-analyses showed that curcumin reduced the incidence of severe R/CIOM (RR 0.42 [0.24, 0.75]) and the mean severity of R/CIOM (MD -0.93 [−1.34, −0.52]). Eleven core target genes were identified in the treatment of R/CIOM with curcumin. The results of molecular docking and the molecular dynamics simulation showed that curcumin had strong binding energy and stability with target proteins including MAPK3, SRC, and TNF. Overall, these findings suggest curcumin can effectively improve severe R/CIOM, perhaps by affecting MAPK3, SRC, and TNF.

## 1. Introduction

Radiation/chemotherapy-induced oral mucositis (R/CIOM) is a frequently encountered and potentially severe adverse effect of oncological radiotherapy for head and neck neoplasms. The prevalence of R/CIOM is substantial, with an estimated 80% of patients subjected to head and neck irradiation encountering this condition, and a considerable proportion—exceeding 50%—experiencing the severe forms classified as grade 3–4 oral mucositis [1]. This iatrogenic complication not only diminishes the quality of life for affected patients but also poses significant challenges to the integrity and efficacy of the radiotherapy program. Furthermore, it can have a profound and adverse impact on the patients’ clinical outcomes and overall survival prognosis [2]. The Mucositis Study Group of the Multinational Association of Supportive Care in Cancer/International Society of Oral Oncology (MASCC/ISOO) has issued clinical guidelines for oral mucositis management, recommending benzydamine mouthwash, honey, and oral glutamine for radiotherapy patients [3,4]. Despite a wide range of explored treatments, no FDA-approved therapy exists specifically for R/CIOM [5].

In recent years, there has been a surge of interest in natural products for the prevention and treatment of radiation/chemotherapy-induced oral mucositis (R/CIOM). This interest stems from their multifaceted properties, which include free radical scavenging, antioxidant, antimicrobial, anti-inflammatory, wound healing, radioprotective, and immunostimulatory effects [6].

Curcumin, with the chemical structure 1,7-bis (4-hydroxy-3-methoxyphenyl)-1,6-heptadiene-3,5-dione, is the predominant bioactive component extracted from the rhizomes of Curcuma longa Linn (Zingiberaceae family), such as turmeric [7]. This compound has a venerable history of use in both the culinary and medicinal realms and has been shown to possess a wide array of biological activities, such as antioxidant, anti-inflammatory, antifungal, antibacterial, and anticancer effects [8]. Several studies have documented the beneficial impact of curcumin on R/CIOM, demonstrating its efficacy in mitigating pain and diminishing associated symptoms [9].

Harnessing curcumin’s therapeutic potential, this study initially confirms the efficacy of curcumin in treating R/CIOM through a meta-analysis. Building upon the recognized clinical benefits of curcumin, we have further employed advanced methodologies, including network pharmacology, molecular docking, and a molecular dynamics simulation, to delve into the molecular underpinnings of curcumin’s therapeutic impact on R/CIOM. This comprehensive approach is designed to lay the scientific groundwork that may accelerate the clinical development and utilization of curcumin.

## 2. Materials and Methods

### 2.1. Protocol and Registration

The protocol of this meta-analysis and systematic review was registered on PROSPERO (CRD42023410826).

### 2.2. Search Strategy

Electronic searches were conducted in the PubMed, Embase, CENTRAL, and Web of Science databases up to 28 May 2023, with additional screening of cited references. The PubMed search strategy is outlined in Supplementary Materials (Table S1).

### 2.3. Selection Criteria

This systematic review includes randomized controlled trials (RCTs) examining the effects of curcumin on radiotherapy (RT)- and/or chemotherapy (CT)-induced oral mucositis (OM) in patients with head and neck cancers. Exclusions encompass nonrandomized studies, conference papers, reviews, abstracts, case reports, case series, and animal or cellular studies, as well as articles without relevant outcomes. The selection process involved three stages: duplicate removal, exclusion of irrelevant papers, and application of defined screening criteria. Two reviewers independently evaluated articles for eligibility, with senior reviewer consultation for disagreements. The criteria for inclusion and exclusion are summarized in Supplementary Materials (Table S2).

### 2.4. Risk of Bias Assessment

Two reviewers independently assessed the risk of bias in the included RCTs using the Cochrane Collaboration tool, focusing on five key domains: the randomization process, intervention adherence, data completeness, outcome measurement, and reporting selectivity [10]. The overall risk of bias for each RCT was determined based on the following criteria: low risk of bias (all domains with a low risk), unclear risk of bias (one or more domains with an unclear risk alongside other domains with a low risk), and high risk of bias (one or more domains with a high risk).

### 2.5. Data Extraction

Author (year), study design, patients, sample size, age, intervention, outcomes, and conclusions were independently extracted by two reviewers. Any disparities in the extraction results were reconciled through consensus with an additional senior reviewer.

#### 2.5.1. Primary Outcomes

1. Incidence of R/CIOM; 2. Incidence of Severe R/CIOM (Grade > 2); 3. Mean R/CIOM Severity.

#### 2.5.2. Secondary Outcomes

1. Oral pain; 2. Dysphagia; 3. Weight Loss.

### 2.6. Data Synthesis

Data analysis was performed using Revman 5.4, with the I^2^ statistic to measure heterogeneity. Given the clinical diversity among trials, such as differences in curcumin formulations and dosages, a random-effects model was applied [11]. Sensitivity analyses were conducted using the “leave one out” approach to assess the impact of individual studies on pooled results. Where a high risk of bias was present, sensitivity analyses excluded these studies. Subgroup analyses were performed based on study characteristics when appropriate. Publication bias was assessed in meta-analyses with a minimum of 10 articles reporting identical outcomes. The overall quality of evidence was evaluated using GRADE profiler 3.6.1 [12].

### 2.7. Target Screening of Curcumin

Details of curcumin (CAS:458-37-7) were sourced from PubChem [13]. All its targets in Homo sapiens were collected from five databases: HERB [14], PharmMapper [15], Targetnet [16] (including only predicted targets with a probability greater than 0), Swiss Target Prediction [17] (including only predicted targets with a probability greater than 0), and SuperPred [18]. All obtained targets were converted into gene names using the UniProt database [19]. Subsequently, the gathered target genes were merged and duplicates were removed.

### 2.8. Gene Screening of R/CIOM Related Targets

OMIM [20], GeneCards [21] (including only genes with relevance score greater than 20), and Disgenet [22] were used to collect relevant targets for “radiation-induced oral mucositis” and “chemotherapy-induced oral mucositis”. Targets obtained from these three disease databases were merged, and duplicates were removed to extract the targets associated with R/CIOM.

### 2.9. Construction of a Protein–Protein Interaction (PPI) Network

The intersection of the target genes of curcumin and the relevant target genes for R/CIOM was plotted on a Venn diagram. The resulting intersection was then submitted to STRING [23] to construct a PPI network.

### 2.10. Go and KEGG Enrichment Analysis

To elucidate the roles of target proteins interacting with curcumin target genes in gene function and signaling pathways, we performed GO and KEGG enrichment analyses using Metascape 3.5 [24]. The obtained results were visualized using the online bioinformatics analysis platform (https://www.bioinformatics.com.cn/ (accessed on 22 January 2014)) [25].

### 2.11. Hub Gene Analysis

The hub genes of the PPI network of curcumin-treated R/CIOM were calculated by the CytoNCA 2.1.6 tool in Cytoscape 3.8.0 [26]. A gene is regarded as a hub gene when its “degree”, “betweenness”, and “closeness” metrics all surpass the average value.

### 2.12. Molecular Docking

Molecular docking was performed between curcumin and the hub genes. The 3D structure of each hub gene’s protein was downloaded from the PDB 2024 [27]. The water molecules and the original ligands were removed from the target proteins through PyMOL2.5.4. Later, the target proteins were imported into AutoDock Tools 1.5.6 for hydrogenation, charge calculation, and non-polar hydrogen combination. Finally, AutoDock Vina was used for molecular docking [28], and PyMOL was employed to visualize the results [29].

### 2.13. Molecular Dynamics Simulation (MD)

This study employed Gromacs v2022.03 software to perform a 100 ns molecular dynamics simulation on the complexes obtained from molecular docking [30,31], using the CHARMM36 force field [32]. The process was as follows: Add the generalized AMBER force field (GAFF) [33] to the small molecule 2 using AmberTools22 software, and calculate the RESP charges for the small molecule with Gaussian 16W, adding it to the molecular dynamics system topology file. Use the three-point transferable intermolecular potential (TIP3P) solvent to dissolve the complex, ensuring that the closest distance from protein atoms to the edge of the water box is at least 1.2 nm (12 Å) [34], and neutralize the simulation system charge by adding an appropriate number of Na^+^ and Cl^-^ (concentration: 0.154 M). Energy minimization (EM) [35] is performed using the steepest descent algorithm to achieve a stable system. Subsequently, the solute is restrained in the isothermal-isobaric (NVT) ensemble, and the system is slowly heated from 0 K to 300 K, then equilibrated at 300 K and 1 Bar pressure in the isothermal–isochoric (NPT) ensemble. A 100 ns time molecular dynamics simulation of the complex is conducted and the simulation trajectory is saved for subsequent analysis. Based on the results of the MD simulation, we calculated the root mean square deviation (RMSD), root mean square fluctuation (RMSF), radius of gyration (Rg) values, solvent-accessible surface area (SASA), and hydrogen bonds (H-bonds) of the complex. Based on the RMSD and Rg values, the Gibbs free energy was calculated using the built-in “g_sham” and “xpm2txt.py” scripts of the Gromacs v2022.03 software. In addition, we applied the “MMPBSA.py v.16.0” script to calculate the binding free energy of the complex using the molecular mechanics/Poisson–Boltzmann surface area (MM/PBSA) method [36].

## 3. Results

### 3.1. Search Results

Initially, 109 titles and abstracts were screened, leading to the selection of 69 papers after duplicate removal. Fifty papers were excluded in the first round for irrelevance, review status, non-human studies, or being protocol papers. The second round considered 19 topic-related papers, excluding one retrospective trial, four without control groups, and one that did not report required outcomes. An additional paper was excluded for using a non-curcumin drug. Full-text review further narrowed down the selection, with reasons detailed in Supplementary Materials (Table S3). In total, twelve studies [37,38,39,40,41,42,43,44,45,46,47,48] were included in the review, as depicted in Supplementary Materials (Figure S1).

### 3.2. Studies Description

This review encompasses twelve RCTs [37,38,39,40,41,42,43,44,45,46,47,48] published between 2014 and 2023, involving a total of 565 patients with head and neck cancers undergoing radiotherapy and/or chemotherapy. Sample sizes varied from 17 to 88 patients, with ages from 30 to 90 years. Four trials involved radiotherapy only [38,39,41,47], one involved chemotherapy only [42], and seven included concurrent radiotherapy and chemotherapy [37,40,43,44,45,46,48]. Intervention durations spanned 2 to 7 weeks, with curcumin administered in forms such as mouthwash, capsules, and gel, with variations in dosage and application methods. For instance, one trial [37] compared different dosages of curcumin capsules, while another [41] evaluated different product forms. The curcumin group comprised 299 patients, and the control group had 266 patients. Further study characteristics are detailed in Table 1.

### 3.3. Risk of Bias

Two RCTs [44,46] were identified with a high risk of bias due to unclear sequence generation, a lack of allocation concealment, and an absence of blinding in the measurement process, which could introduce measurement bias. Another trial [38] was rated high risk due to significant patient dropout. Three trials [39,45,48] raised concerns for insufficient detail on blinding, while two trials [41,42] were noted for inadequate data reporting. Conversely, four trials [37,40,43,47] were low risk, offering full transparency in study design. The risk of bias assessment for all RCTs is detailed in Supplementary Materials (Table S4).

### 3.4. Meta Outcome

#### 3.4.1. Primary Outcomes

**Figure 1:**
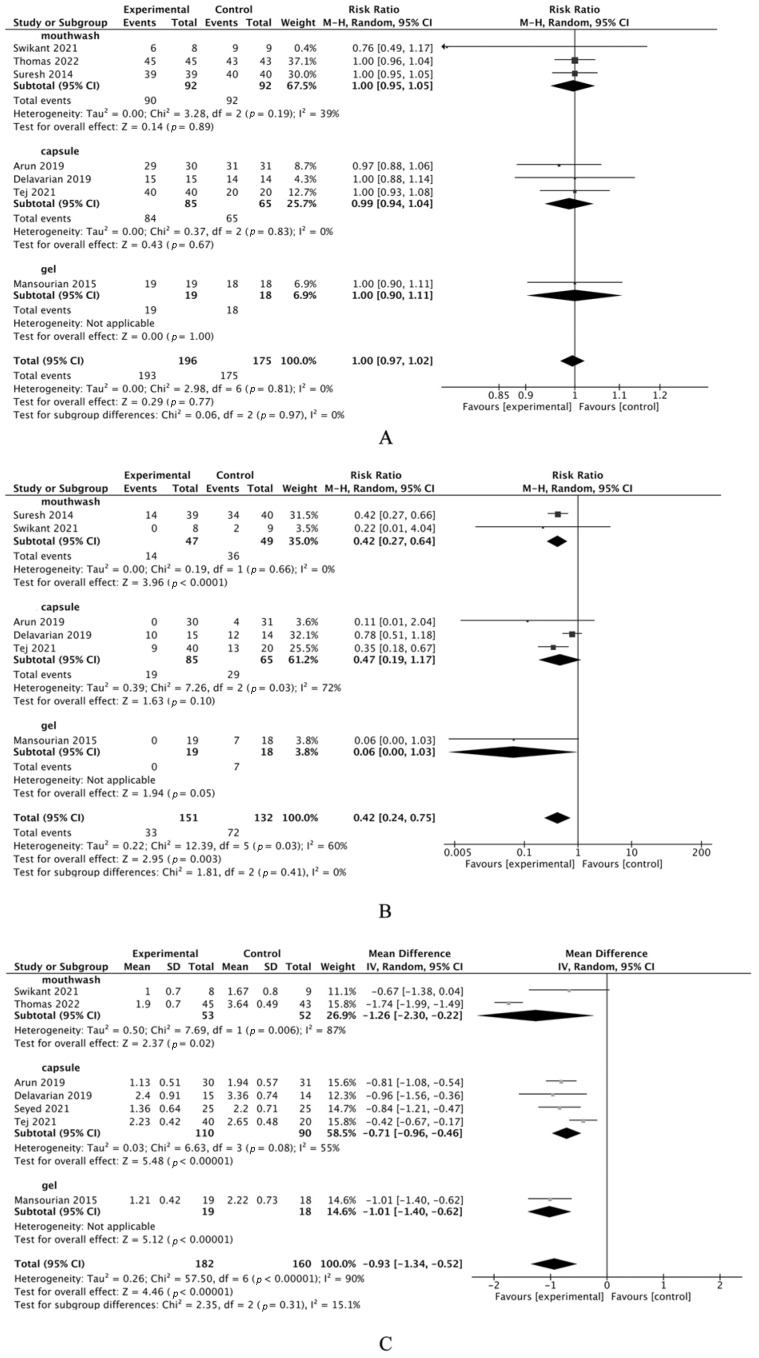
Meta-analysis of primary outcomes of curcumin treatment for R/CIOM. (**A**): Meta-analysis of the incidence of R/CIOM. (**B**): Meta-analysis of the incidence of severe R/CIOM. (**C**): Meta-analysis of the mean severity of R/CIOM. Tej (2021) [37], Swikant (2021) [38], Thomas (2022) [39], Seyed (2021) [40], Suresh (2014) [43], Mansourian (2015) [45], Delavarian. (2019) [47], Arun (2019) [48]. ■: The effect size of the study, in turn, signifies the extent to which the study’s findings contribute to the overall meta-analysis. Meanwhile, the magnitude of the square represents the study’s impact within this synthesis. ◆: The pooled result.

Seven studies [37,38,39,43,45,47,48], with a total of 371 patients, reported on R/CIOM incidence, showing no significant difference between curcumin and placebo groups in a meta-analysis (risk ratio 1.00, 95%CI [0.97 to 1.02]; *p* = 0.77; Figure 1A), with no observed heterogeneity (I^2^ = 0%). Six studies [37,38,43,45,47,48], with a total of 283 patients, indicated a significantly lower risk of severe R/CIOM (Grade > 2) in the curcumin group (risk ratio 0.42, 95%CI [0.24 to 0.75]; *p* = 0.003; Figure 1B), though with moderate heterogeneity (I^2^ = 60%). Analysis of mean R/CIOM severity from seven trials [37,38,39,40,45,47,48] involving 342 patients revealed significantly less severity in the curcumin group (pooled MD = −0.93, 95%CI [−1.34 to −0.52], *p* < 0.00001, Figure 1C), but with substantial heterogeneity (I^2^ = 90%). Comprehensive details are in Table 2.

#### 3.4.2. Secondary Outcomes

**Figure 2:**
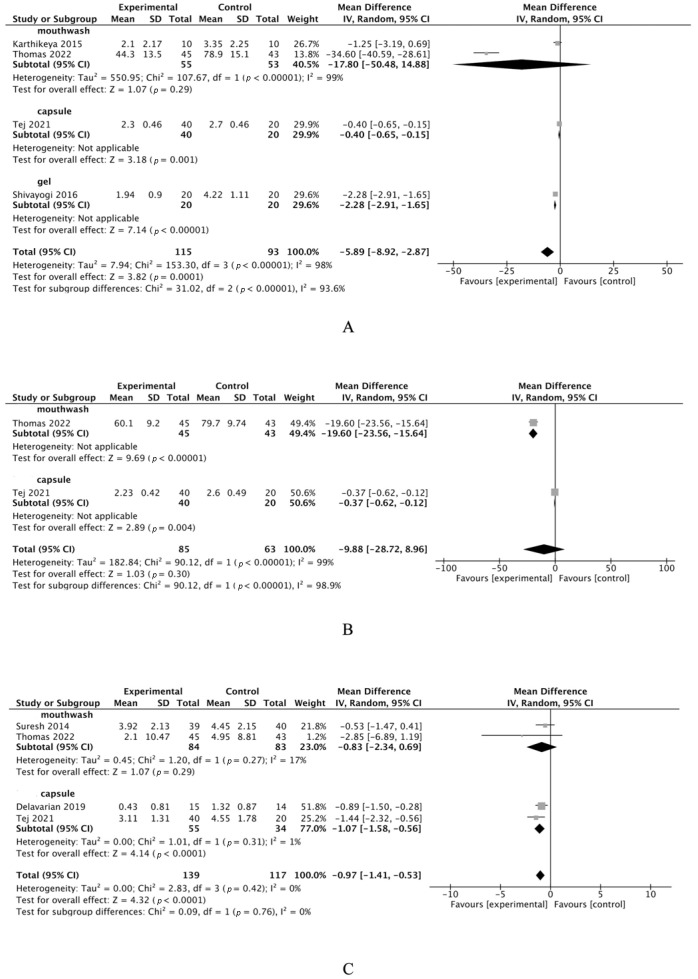
Meta-analysis secondary of curcumin treatment for R/CIOM. (**A**): Meta-analysis of the oral pain in R/CIOM. (**B**): Meta-analysis of the dysphagia in R/CIOM. (**C**): Meta-analysis of the weight loss in R/CIOM. Tej (2021) [37], Swikant (2021) [38], Thomas (2022) [39], Suresh (2014) [43], Karthikeya (2015) [44], Shivayogi (2016) [46], Delavarian (2019) [47]. ■: The effect size of the study, in turn, signifies the extent to which the study’s findings contribute to the overall meta-analysis. Meanwhile, the magnitude of the square represents the study’s impact within this synthesis. ◆: The pooled result.

The analysis results of oral pain, dysphagia, and weight loss are displayed in Table 2 and Figure 2. The meta-analysis evidenced a significant reduction in oral pain and weight loss among the curcumin-treated group compared to the placebo cohort.

#### 3.4.3. Sensitivity Analyses

Sensitivity analysis was conducted to evaluate the influence of high-risk-of-bias studies on meta-analysis outcomes. The three datasets—R/CIOM incidence, severe R/CIOM incidence (Grade > 2), and mean R/CIOM severity—each initially included one high-risk article [38]. For oral pain, two high-risk articles [44,46] were factored in. Excluding these, the analysis showed stable results across all datasets except for oral pain. Using the “leave one out” method, I^2^ values significantly decreased for severe R/CIOM incidence and mean R/CIOM severity when articles solely involving radiotherapy patients were removed. Further details are available in Table 2.

#### 3.4.4. Subgroup Analyses

Subgroup analyses were performed due to the diversity of curcumin products in the studies, with results summarized in Table 3.

#### 3.4.5. GRADE Assessment

Evidence quality was rated as high for R/CIOM incidence and weight loss, moderate for severe R/CIOM incidence (Grade > 2), low for mean R/CIOM severity and dysphagia, and very low for oral pain. Downgrading primarily resulted from risk of bias and inconsistency. Four outcomes showed moderate to high heterogeneity and included high-risk-of-bias articles. Dysphagia was downgraded due to the small sample size. No indirectness-related downgrades occurred, while two outcomes were considered for upgrading due to significant effect size. Details are in Table 4.

### 3.5. Related Targets of Curcumin and R/CIOM

After screening and merging, there was a total of 662 potential target genes for curcumin, and 521 genes related to R/CIOM. Subsequent intersection analysis revealed a subset of 131 genes common to both categories.

### 3.6. PPI Network

**Figure 3:**
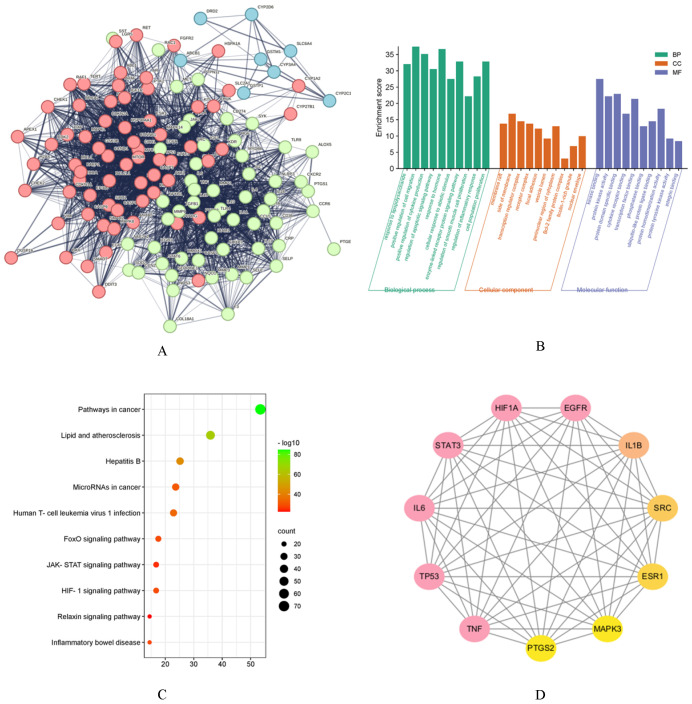
The results of network pharmacology. (**A**): PPI network. (**B**): Go enrichment. (**C**): KEGG enrichment. (**D**): Hub gene.

A total of 131 co-expressed genes were subjected to PPI analysis, revealing networks consisting of 129 nodes and 4016 edges (Figure 3A). The PPI enrichment *p*-value was <1.0 × 10^−16^.

### 3.7. GO Analysis and KEGG Analysis Results

Functional analysis through gene ontology (GO) suggested that curcumin potentially triggers positive regulation of cell migration within biological processes and displays kinase binding in molecular functions, illustrated in Figure 3B. Moreover, the KEGG enrichment analysis revealed that the co-expressed genes were primarily associated with pathways related to cancer and other biological functions. Figure 3C depicts the top 10 pathways identified in this analysis.

### 3.8. Hub Gene Analysis

The hub genes within the PPI network of curcumin-treated R/CIOM were calculated by the CytoNCA tool in Cytoscape 3.8.0. The average values of “degree”, “betweenness”, and “closeness” were 62.26, 66.80, and 0.67, respectively. The identified hub genes were PTGS2, MAPK3, ESR1, SRC, IL1B, EGFR, HIF1A, STAT3, IL6, TP53, and TNF (Figure 3D).

### 3.9. Molecular Docking

**Figure 4:**
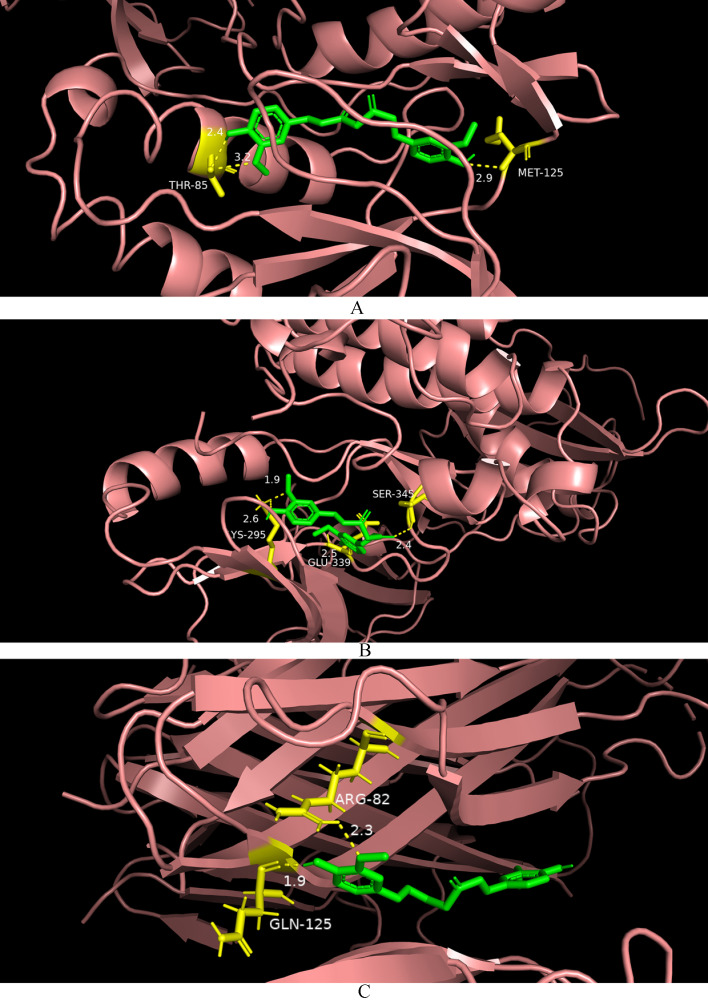
The results of molecular docking. (**A**): curcumin docks with MAPK3. (**B**): curcumin docks with SRC. (**C**): curcumin docks with TNF.

In order to validate the network pharmacology findings, molecular docking was utilized to assess the binding affinity between curcumin and the hub genes. The binding energies are presented in Table 5, while Figure 4 displays the docking outcomes showing strong binding activity (below −8 kcal/mol).

### 3.10. MD

**Figure 5:**
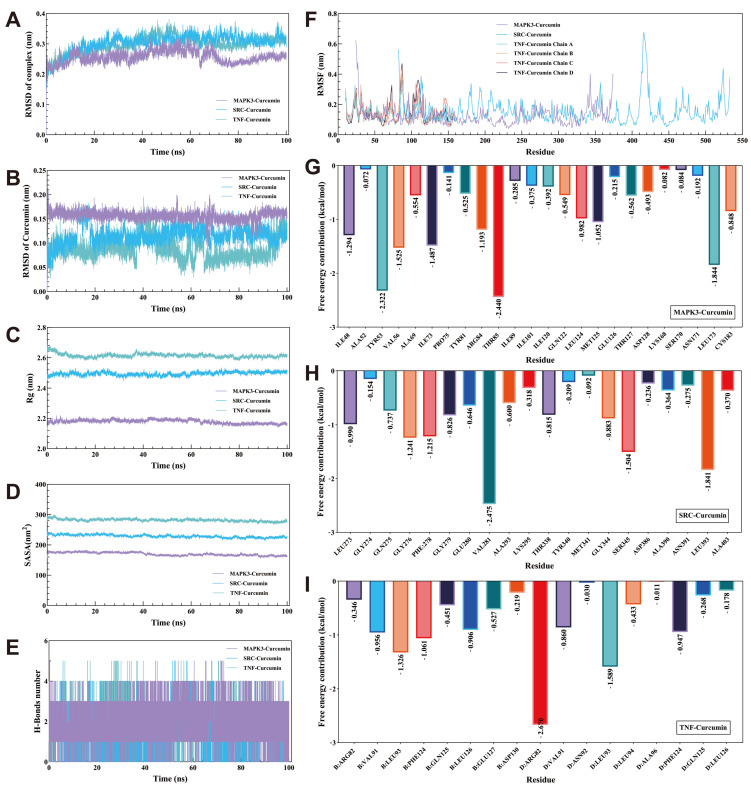
The results of the molecular dynamics simulation. (**A**): RMSD curve of the MAPK3/SRC/TNF–curcumin complex. (**B**): RMSD curve of curcumin. (**C**): Rg curve of the MAPK3/SRC/TNF–curcumin complex. (**D**): SASA curve of the MAPK3/SRC/TNF–curcumin complex. (**E**): Number of hydrogen bonds in the MAPK3/SRC/TNF–curcumin complex. (**F**): RMSF curve of MAPK3/SRC/TNF. (**G**–**I**): Amino acid decomposition of the MAPK3/SRC/TNF–curcumin complex.

**Figure 6:**
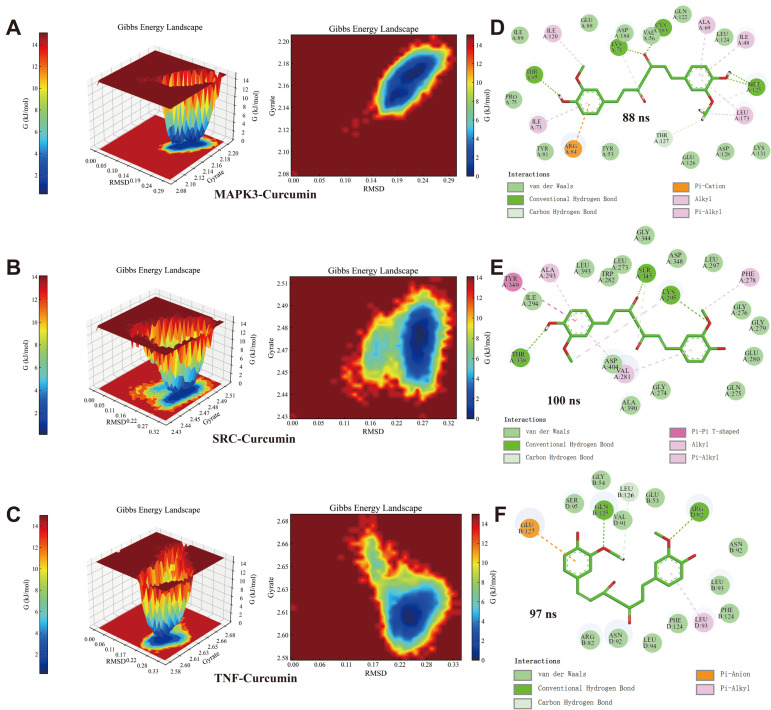
The results of the Gibbs free energy analysis. (**A**–**C**): 3D and 2D contour plots of the MAPK3/SRC/TNF–curcumin complex. (**D**–**F**): 2D interaction maps at the lowest Gibbs free energy state of the MAPK3/SRC/TNF–curcumin complex.

The RMSD curves for the MAPK3/SRC/TNF–curcumin complexes showed equilibrium after 76 ns, with average RMSD values of 0.25 nm, 0.32 nm, and 0.3 nm (Figure 5A). Stability assessments through RMSD calculations relative to the initial structure (Figure 5B) indicated the least positional deviation for curcumin when bound to MAPK3 among the three complexes. The Rg values for these complexes-maintained equilibrium with means of 2.16 nm, 2.5 nm, and 2.6 nm (Figure 5C). The corresponding SASA values were stable at averages of 270 nm^2^, 230 nm^2^, and 280 nm^2^ (Figure 5D). During the 100 ns simulation, hydrogen bond numbers for the MAPK3/SRC/TNF–curcumin complexes ranged from 2–5, 2–5, and 1–5, respectively (Figure 5E). RMSF analysis identified key flexible regions in MAPK3, SRC, and TNF proteins, specifically around amino acid residues ILE48, TYR53, VAL56, ILE73, ARG84, THR85, LEU173 for MAPK3–curcumin; GLY276, PHE278, VAL281, SER345, LEU393 for SRC–curcumin; and LEU93 (B chain), PHE124 (B chain), ARG82 (D chain), LEU93 (D chain) for TNF–curcumin (Figure 5F–I). The Gibbs free energy profiles for the complexes showed distinct minimum energy wells (Figure 6A–C), with hydrogen bond counts at 5, 3, and 2, respectively (Figure 6D–F). The binding free energy data for the MAPK3/SRC/TNF–curcumin complexes are presented in Table 6.

## 4. Discussion

Our meta-analysis shows curcumin significantly reduces severe R/CIOM incidence and helps alleviate pain and weight loss. Following this, we preliminarily explored curcumin’s mechanism through network pharmacology, molecular docking, and a molecular dynamics simulation, identifying MAPK3, SRC, and TNF as key target proteins for its effects.

Our updated meta-analysis includes 12 RCTs, surpassing the previous one [49], and has assessed the evidence level, reinforcing curcumin’s potential as an R/CIOM treatment. We also broadened the scope to include pain, dysphagia, and weight loss—key R/CIOM symptoms—to better gauge curcumin’s therapeutic efficacy. With the exception of the overall incidence of R/CIOM, some heterogeneity was observed in some of the meta-analysis outcomes. This heterogeneity is likely due to inconsistencies across studies in the types of curcumin formulations used (such as mouthwash, capsules, and gel), as well as variations in dosage and treatment duration. Despite the current lack of standardization in dosage, duration, and formulation for the clinical use of curcumin to treat R/CIOM, our study indicates that curcumin significantly reduces the incidence and severity of severe R/CIOM and also alleviates associated symptoms such as pain, dysphagia, and weight loss. Studies even suggest that curcumin/turmeric mouthwash may outperform the benzydamine mouthwash endorsed by MASCC/ISOO [50] in mitigating oral mucositis and dysfunctions [39], or in delaying the onset of R/CIOM [38]. Moreover, daily curcumin consumption positively modulates the body’s inflammatory response [51] and is associated with minimal side effects [52]. However, the clinical application of natural curcumin is limited by its hydrophobicity, low gastric absorption rate, photosensitivity, and low bioavailability [53]. To address these challenges, recent research has concentrated on formulating curcumin in ways that enhance its bio-efficacy. For instance, the bio-enhanced turmeric formulation (BTF), which combines curcumin with curcuminoids, has shown effectiveness in reducing the severity of oral mucositis, dysphagia, oral pain, and dermatitis induced by chemoradiotherapy [37]. Nanosizing curcumin is another approach that has demonstrated positive effects on R/CIOM treatment in both mouthwash [38] and capsule [40,41,47] forms. However, one study [41] found no significant difference in the effectiveness of nanocurcumin capsules and regular curcumin mouthwash for R/CIOM treatment. Further research is warranted to identify the optimal curcumin treatment strategies for R/CIOM.

Research indicates that curcumin demonstrates effective treatment potential for various inflammatory conditions, such as radiation dermatitis [54], pneumonia [55], and mucositis/enteritis [56], attributed to its anti-inflammatory properties [57]. Effectively addressing R/CIOM hinges on managing the escalation of the inflammatory response. In the pathogenesis of R/CIOM, chemoradiotherapy initiates cellular and tissue damage, leading to reactive oxygen species (ROS) production. Consequently, ROS signaling upregulates proinflammatory factors, intensifying and expediting inflammatory and immune signals. Ultimately, mucosal damage occurs, culminating in ulcer formation [58]. Based on the potent anti-inflammatory attributes of curcumin [59], through further network pharmacology analysis, this study identified potential therapeutic targets of curcumin for treating R/CIOM, including PTGS2, MAPK3, ESR1, SRC, IL1B, EGFR, HIF1A, STAT3, IL6, TP53, and TNF. Among them, MAPK3, SRC, and TNF exhibit the strongest binding activity. In our molecular dynamics simulation, analyses including RMSD, RMSF, Rg, SASA, and Gibbs free energy all indicate that curcumin can form stable complexes with MAPK3, SRC, and TNF proteins. This provides solid theoretical support for the biological functions of curcumin.

Mitogen-activated protein kinase 3 (MAPK3), also known as extracellular signal-regulated kinase 1 (ERK1), plays a pivotal role in the ERK/MAPK pathway, regulating apoptosis, cell proliferation, and migration [60]. It is a crucial component involved in the phosphorylation and translocation of various cytosolic proteins into the nucleus, contributing significantly to inflammatory processes [61]. Although there is limited research on the ERK/MAPK pathway in R/CIOM, studies have associated this pathway with the healing of skin wounds [61] and gastric ulcers [62]. However, the precise mechanisms underlying the impact of the ERK/MAPK pathway on wound healing remain unclear. Studies suggest that activating ERK1/2 could increase MMP9 (matrix metalloproteinase 9) expression, leading to delayed wound healing in conditions like diabetic foot ulcers [61]. Conversely, inhibiting the phosphorylation activation of ERK1/2 has been demonstrated to impede gastric epithelial cell proliferation and angiogenesis, thereby slowing the healing of gastric ulcers [62,63]. Current research has extensively explored the interplay between curcumin and ERK in various diseases. However, the specific mechanisms through which curcumin influences ERK1 remain elusive. For instance, curcumin has been reported to suppress proliferation and induce apoptosis in human placental choriocarcinoma cells through the activation of ERK1/2 [64]. Additionally, curcumin has shown promising results in attenuating myocardial injury by suppressing ERK1/2 [65] but expediting the repair of sciatic nerve injuries in rats through the activation of ERK1/2 [66]. Therefore, the precise impact of curcumin on ERK1 in the context of treating R/CIOM requires further investigation for conclusive understanding.

As for SRC, the SRC family comprises non-receptor tyrosine kinases that are highly expressed in epithelial cells and susceptible to activation by ROS [67]. SRC initiates the EGFR signaling cascade [68] and the JNK signaling pathway [69], contributing to an inflammatory response. Studies have indicated the effectiveness of inhibiting inflammation through the suppression of EGFR signaling [70] and JNK signaling [71]. Animal research has demonstrated that Laminaria japonica polysaccharides can inhibit JNK activation, thereby mitigating radiation-induced damage to the salivary glands [72]. Early investigations revealed that curcumin could inhibit SRC activation, consequently retarding cellular growth and migration [73]. Additionally, nanospheres loaded with curcumin (CN) were shown to inhibit the phosphorylation of c-Src [74]. Research has demonstrated that curcumin effectively alleviates gingival overgrowth by inhibiting the phosphorylations of SRC and JNK [75], and it also enhances the therapeutic effects of chemotherapy against colorectal cancer cells [76]. Further understanding how curcumin influences R/CIOM through the modulation of SRC is crucial for the meaningful application of curcumin in R/CIOM treatment.

Tumor necrosis factor (TNF) is a crucial cytokine in causing damage in R/CIOM. As a pro-inflammatory cytokine, TNF initiates the activation of MAPK on target cells and concurrently sustains NF-κB activity. Consequently, this process leads to damage in connective tissue and endothelium, inhibits tissue oxygenation, and promotes the death of epithelial basal cells [77]. Studies have even found that TNF can serve as a monitoring marker for the severity of R/CIOM, where patients with higher levels of TNF tend to exhibit more severe oral mucositis [78,79]. Research has demonstrated that benzydamine [80] and pentoxifylline [81], acting as anti-inflammatory agents by inhibiting TNF, have beneficial therapeutic effects on R/CIOM. Curcumin, in particular, exerts its anti-inflammatory effects by reducing TNF and suppressing post-inflammatory pathways [82,83,84]. This may be a crucial mechanism through which curcumin plays a therapeutic role in R/CIOM.

## 5. Conclusions

In summary, this study underscores the significant potential of curcumin in not only reducing the incidence of severe R/CIOM, characterized by a grade greater than 2, but also in alleviating the intensity of associated symptoms such as pain and weight loss. Curcumin’s multifaceted interaction with key molecular targets, including MAPK3, SRC, and TNF, is suggested to be the driving force behind its observed anti-inflammatory effects. These interactions are crucial in modulating the body’s response to inflammation, which is a key factor in the development of R/CIOM.

## Figures and Tables

**Table 1:** Summary of findings for the 12 studies included within this review.

	Author	Study Design	Participants	Sample Size (F/M)	Age	Intervention Technique	Observation Cycle	Outcomes	Conclusion
1	Suresh Rao et al. (2014) [43]	RCT	Patients of head and neck cancer scheduled to receive radiotherapy or chemoradiotherapy.	79 (NA) (E:39, C:40)	Mean 55.96 ± 12.25 years	Experiment: turmeric mouthwash (400 mg Turmeric capsule dissolved in approximately 80 mL of boiled and cooled water), 6 times/day Control: povidone–iodine solution (diluted 1:100), 2 times/day	7 weeks	1. Incidence of R/CIOM 2. Incidence of severe R/CIOM (Grade > 2) 3. Weight loss	Gargling with turmeric by head and neck cancer patients undergoing radiation therapy provided significant benefit by delaying and reducing the severity of mucositis. Turmeric is readily available, relatively inexpensive, and highly accepted, making it useful in cancer treatment.
2	Karthikeya Patil et al. (2015) [44]	RCT	Head and neck cancer patients undergoing radio-chemotherapy	20 (9/11) (E:10, C:10)	39–71 years	Experiment: curcumin mouthrinse 0.004%, (diluted 1:5), 3 times/day Control: chlorhexidine 0.2% (diluted 1:1), 3 times/day	20 days	1. oral pain 2. The score for erythema 3. The score for ulcerations	Curcumin was found to be better than chlorhexidine mouthwash in terms of rapid wound healing and better patient compliance in management of radio-chemotherapy-induced oral mucositis. No oral or systemic complications were reported.
3	Mansourian et al. (2015) [45]	RCT	Patients with head and neck cancer admitted for radiation therapy	37 (31/6) (E:19, C:18)	Mean 51.34 ± 12.86 years	Experiment: curcuma longa topical gel (dried hydroalcohol derivative of curcuma Longa, 0.5% gel), 3 times/day Control: a yellowish ineffective inert placebo in the form of gel, 3 times/day	3 weeks	1. Incidence of R/CIOM 2. Incidence of severe R/CIOM (Grade > 2) 3. Mean R/CIOM severity 4. The max size of erythema 5. The max size of ulcer	A topical gel, containing curcuma longa’s derivate, can effectively reduce the oral symptoms of mucositis in patients undergoing head and neck cancer radiotherapy
4	Shivayogi Charantimath et al. (2016) [46]	RCT	Oral cancer patients undergoing radiotherapy or chemoradiotherapy.	40 (3/37) (E:20, C:20)	NA	Experiment: curcuma gel (per gram of gel contained 10 mg of Curcuma longa extract), 3 times/day Control: chlorhexidine gluconate 1.0%, 3 times/day	2 weeks	1. Oral pain 2. The score for erythema 3. The score for ulcerations	Curcumin gel appears to be an effective and safer alternative tochlorhexidine gel in treatment of oral mucositis.
5	Delavarian et al. (2019) [47]	RCT	Patients with head and neck cancer undergoing radiotherapy	29 (NA) (E:15, C:14)	Mean 59.03 ± 15.29 years	Experiment: oral nanocurcumin (1 capsule of SinaCurcumin ^®^80 mg/day) Control: placebo tablets (containing lactose, 1 tablets/day).	6 weeks	1. Incidence of R/CIOM 2. Incidence of severe R/CIOM (Grade > 2) 3. Mean R/CIOM severity 4. Weight loss	Nanomicelle curcumin is an effective agent in the prevention of OM or reducing its severity
6	Arun et al. (2019) [48]	RCT	Patients with head and neck cancer undergoing post-operative radiotherapy, post-operative chemoradiotherapy, or concurrent chemoradiotherapy	61 (33/28) (E:30, C:31)	30–90 years	Experiment: 500 mg of turmeric extract capsules (BCM-95 ^®^/Curcugreen ^®^), 3 times/day Control: placebo capsule containing starch powder, 3 times/day	4 weeks	1. Incidence of R/CIOM 2. Incidence of severe R/CIOM (Grade > 2) 3. Mean R/CIOM severity	Turmeric extract reduces the incidence and severity of radiation-induced mucositis, which can benefit patients undergoing radiation for head and neck cancer.
7	Tej et al. (2021) [37]	RCT	Patients who had undergone radical surgery (wide excision with modified neck dissection) for oral cavity cancer undergoing radio-chemotherapy	60 (5/55) (E:40, C:20)	Mean 43.67 ± 2.62 year	Experiment A: 500 mg Bio-enhanced turmeric formulation capsules (low dose [1 g/day]) Experiment B: bio-enhanced turmeric formulation capsules high dose [1.5 g/day]) Control: placebo capsules, 3 times/day	6 weeks	1. Incidence of R/CIOM 2. Incidence of severe R/CIOM (Grade > 2) 3. Mean R/CIOM severity 4. Oral pain 5. Dysphagia 6. Weight loss	A bio-enhanced turmeric formulation can significantly reduce chemoradiotherapy-induced severe oral mucositis, dysphagia, oral pain, and dermatitis in oral cancer patients.
8	Swikant et al. (2021) [38]	RCT	Head and neck cancer patients scheduled to receive radiation therapy	17 (NA) (E:8, C:9)	Mean 54.34 ± 13.78 years	Experiment: 0.1% curcumin (freshly prepared using nanoparticles) mouthwash, 3 times/day Control:0.15% benzydamine mouthwash, 3 times/day	6 weeks	1. Incidence of R/CIOM 2. Incidence of severe R/CIOM (Grade > 2) 3.Mean R/CIOM severity	Use of 0.1% curcumin mouthwash was able to significantly delay the onset of radiation-induced oral mucositis.
9	Seyed et al. (2021) [40]	RCT	Patients undergoing chemotherapy with or without head and neck radiotherapy	50 (21/29) (E:25, C:25)	Mean 55.96 ± 1.10 years	Experiment: curcumin nanomicelle capsules, 80 mg 2 times/day Control: placebo capsules, 2 times/day	7. weeks	1. Mean R/CIOM severity 2. Oral pain	Nabomicelle curcumin capsules is effective on prevention and treatment of head and neck radiotherapy and especially chemotherapy-induced oral mucositis.
10	Thomas (2022) [39]	RCT	Adult patients with head and neck cancer receiving radiation therapy	88 (NA) (E:45, C:43)	Mean 57.60 ± 11.64 years	Experiment: turmeric mouthwash (dissolving the contents of the 400 mg turmeric capsule in 80 mL of boiled cooled water), 6 times/day Control: benzydamine mouthwash, 6 times/day	7 weeks	1. Incidence of R/CIOM 2. Mean R/CIOM severity 3. Oral pain 4. Dysphagia 5. Weight loss	Turmeric mouthwash was effective in reducing the severity of oral mucositis and associated oral dysfunctions as compared with benzydamine mouthwash
11	Farshid et al. (2023) [42]	RCT	Cancer patients with chemotherapy-induced oral mucositis	47 (34/13) (E:23, C:24)	Mean 58.83 ± 13.33 years	Experiment: curcumin gel 0.5%, 4 times/day Control: oral chlorhexidine mouthrinse 0.2%, 4 times/day	2 weeks	1. The score for erythema 2. The score for ulcerations	Curcumin could result in faster recovery in comparison with mucosamin and chlorhexidine. The use of curcumin in the treatment of oral mucositis appears to be a viable intervention for reducing potential compromise to treatment and improving the quality of life.
12	Vahid Ramezani et al. (2023) [41]	RCT	Patients with head and neck cancer with radiotherapy-induced oral mucositis	37 (14/23) (E:25, C:12)	Mean 53.36 ± 15.99 years	Experiment A: curcumin mouthwash (0.1% *w*/*v*), 3 times/day Experiment B: SinaCurcumin ^®^40 (containing 40 mg curcuminoids), one capsule of SinaCurcumin ^®^40/day Control: placebo mouthwash, 3 times/day	3 weeks	1. Mean R/CIOM severity 2. Oral pain	Both curcumin mouthwash and nanocapsules were effective, safe, and well-tolerated in the treatment of radiation-induced oral mucositis.

**Table 2:** The results of sensitivity analyses.

	Incidence of R/CIOM	Incidence of Severe R/CIOM (Grade > 2)	Mean R/CIOM Severity	Oral Pain	Weight Loss	Dysphagia
Pooled	1.00 [0.97, 1.02] (I^2^ = 0%)	0.42 [0.24, 0.75] (I^2^ = 60%)	−0.93 [−1.34, −0.52] (I^2^ = 90%)	−5.89 [−8.92, −2.87] (I^2^ = 98%)	−0.97 [−1.41, −0.53] (I^2^ = 0%)	−9.88 [−28.72, 8.96] (I^2^ = 99%)
Excluding						
articles with high risk of bias	1.00 [0.97, 1.02] (I^2^ = 0%)	0.43 [0.24, 0.78] (I^2^ = 66%)	−0.96 [−1.41, −0.52] (I^2^ = 91%)	−17.36 [−50.88, 16.15] * (I^2^ = 99%)	NA	NA
Suresh Rao et al. (2014) [43]	0.99 [0.96, 1.03] (I^2^ = 0%)	0.35 [0.13, 0.93] (I^2^ = 70%)	NA	NA	−1.10 [−1.60, −0.60] (I^2^ = 0%)	NA
Karthikeya Patil et al. (2015) [44]	NA	NA	NA	−7.86 [−11.56, −4.16] (I^2^ = 99%)	NA	NA
Mansourian et al. (2015) [45]	-	0.47 [0.28, 0.78] (I^2^ = 54%)	−0.92 [−1.39, −0.44] (I^2^ = 91%)	NA	NA	NA
Shivayogi Charantimath et al. (2016) [46]	NA	NA	NA	−11.01 [−19.68, −2.34] (I^2^ = 98%)	NA	NA
Delavarian et al. (2019) [47]	-	0.37 [0.26, 0.54] (I^2^ = 0%) ^&^	−0.93 [−1.38, −0.47] (I^2^ = 91%)	NA	−1.08 [−1.88, −0.27] (I^2^ = 25%)	NA
Arun et al. (2019) [48]	1.00 [0.97, 1.03] (I^2^ = 0%)	0.45 [0.26, 0.79] (I^2^ = 62%)	−0.95 [−1.46, −0.45] (I^2^ = 91%)	NA	NA	NA
Tej et al. (2021) [37]	-	0.43 [0.20, 0.91] (I^2^ = 64%)	−1.03 [−1.43, −0.64] (I^2^ = 85%)	−11.65 [−20.19, −3.10] (I^2^ = 98%)	−0.82 [−1.33, −0.31] (I^2^ = 0%)	NA
Swikant et al. (2021) [38]	-	0.43 [0.24, 0.78] (I^2^ = 66%)	−0.96 [−1.41, −0.52] (I^2^ = 91%)	NA	NA	NA
Seyed et al. (2021) [40]	NA	NA	−0.95 [−1.42, −0.47] (I^2^ = 91%)	NA	NA	NA
Thomas (2022) [39]	0.99 [0.96, 1.03] (I^2^ = 0%)	NA	−0.75 [−0.97, −0.54] (I^2^ = 46%) ^&^	−1.30 [−2.81, 0.21] * (I^2^ = 93%)	−0.95 [−1.39, −0.51] (I^2^ = 0%)	NA

*: Significantly different from the original pooled results; NA, not included the related articles; -, same as the original pooled outcome; &: Significantly decreased from the original I^2^.

**Table 3:** The results of subgroup analyses.

Curcumin Product	Incidence of R/CIOM	Incidence of Severe R/CIOM (Grade > 2)	Mean R/CIOM Severity	Oral Pain	Weight Loss	Dysphagia
Pooled	1.00 [0.97, 1.02]	0.42 [0.24, 0.75]	−0.93 [−1.34, −0.52]	−5.89 [−8.92, −2.87]	−0.97 [−1.41, −0.53]	−9.88 [−28.72, 8.96]
Mouthwash	1.00 [0.95, 1.05]	0.42 [0.27, 0.64]	−1.26 [−2.30, −0.22]	−17.80 [−50.48, 14.88] *	−0.83 [−2.34, 0.69] *	−19.60 [−23.56, −15.64] #
Capsule	0.99 [0.94, 1.04]	0.47 [0.19, 1.17] *	−0.71 [−0.96, −0.46]	−0.40 [−0.65, −0.15] #	−1.07 [−1.58, −0.56]	−0.37 [−0.62, −0.12] #
Gel	1.00 [0.90, 1.11] #	0.06 [0.00, 1.03] #	−1.01 [−1.40, −0.62] #	−2.28 [−2.91, −1.65] #	NA	NA

* Different from the pooled results; # Only one article; NA, not available.

**Table 4:** Evidence profile of outcomes using GRADE assessment.

Quality Assessment							Number of Patients		Effect		Quality
Outcomes	Number of Studies	Risk of Bias	Inconsistency	Indirectness	Imprecision	Other Considerations	Curcumin	Control	Relative	Absolute	
Incidence of R/CIOM	7RCTs	Serious ^1^	Not serious	Not serious	Not serious	Large effect ^2^	196	175	RR 1.00 [0.97, 1.02]	-	⊕⊕⊕⊕ High
Incidence of severe R/CIOM (Grade > 2)	6RCTs	Serious ^1^	Serious ^3^	Not serious	Not serious	Large effect ^4^	151	132	RR 0.42 [0.24, 0.75]	-	⊕⊕⊕⊝ Moderate
Mean R/CIOM severity	7RCTs	Serious ^1^	Serious ^5^	Not serious	Not serious	None	174	151	-	MD −0.93 [−1.34, −0.52]	⊕⊕⊝⊝ Low
Oral pain	4RCTs	Very serious ^6^	Serious ^7^	Not serious	Not serious	None	115	93	-	MD −5.89 [−8.92, −2.87]	⊕⊝⊝⊝ Very low
Dysphagia	2RCTs	Not serious	Serious ^8^	Not serious	Serious ^9^	None	85	63	-	MD −9.88 [−28.72, 8.96]	⊕⊕⊝⊝ Low
Weight Loss	4RCTs	Not serious	Not serious	Not serious	Not serious	None	139	117	-	MD −0.97 [−1.41, −0.53]	⊕⊕⊕⊕ High

^1^ One RCT with high risk of bias; ^2^ RR (1.00,95%,0.97 to 1.02) and 0% heterogeneity; ^3^ I^2^ = 60%, moderate heterogeneity was detected among studies; ^4^ RR < 0.5; ^5^ I^2^ = 91%, large heterogeneity was detected among studies; ^6^ Two RCTs with high risk of bias; ^7^ I^2^ = 98%, large heterogeneity was detected among studies; ^8^ I^2^ = 99%, large heterogeneity was detected among studies; ^9^ The total sample size was not large. ⊕⊕⊕⊕: High quality of the evidence, ⊕⊕⊕⊝: Moderate quality of the evidence, ⊕⊕⊝⊝: Low quality of the evidence, ⊕⊝⊝⊝: Very Low quality of the evidence.

**Table 5:** Molecular docking binding energy.

Target	PDB-ID	Binding Energy (kcal·mol^−1^)
PTGS2	5IKR	−7.3
MAPK3	4QTB	−10.2
ESR1	1SJ0	−5.9
SRC	1FMK	−8
IL1B	5R8Q	−7
EGFR	5D41	−7
HIF1A	2CGO	−7.2
STAT3	6NJS	−6.8
IL6	1ALU	−5.4
TP53	4MZI	−5.6
TNF	2AZ5	−8

**Table 6:** Analysis of Binding Free Energy of the MAPK3/SRC/TNF-Curcumin Complex (kcal/mol).

Energy Contributions	MAPK3-Curcumin	SRC-Curcumin	TNF-Curcumin
ΔVDWAALS	−51.85	−46.64	−44.49
ΔE*_elec_*	−20.02	−25.50	−13.57
ΔE*_surf_*	−7.63	−6.92	−6.29
ΔG*_gas_*	−71.87	−72.14	−58.07
ΔG*_solvation_*	27.84	35.75	29.25
ΔG*_Bind_*	−44.02	−36.38	−28.81

## Data Availability

Data will be made available on request.

## Supplementary Materials

The following supporting information can be downloaded at: https://www.mdpi.com/article/10.3390/cimb46090625/s1, Table S1. Search strategy; Table S2. Inclusion and exclusion criteria for the review; Table S3. Studies excluded after full text assessment with corresponding main reason of exclusion; Figure S1. The flowchart of the research selection process; Table S4. Risk of Bias Assessment for RCTs using the Cochrane Risk of Bias Tool 2.0 [85,86,87,88,89,90].
