# Supplementary material for: Effects of Curcumin on Radiation/Chemotherapy-Induced Oral Mucositis: Combined Meta-Analysis, Network Pharmacology, Molecular Docking, and Molecular Dynamics Simulation
Source: Curr Issues Mol Biol. 2024 Sep 20;46(9):10545–69. doi: 10.3390/cimb46090625 (PMC11431004; doi:10.3390/cimb46090625)
Supplement: Supplementary file 1 [file cimb-46-00625-s001.zip › cimb-3219930-supplementary-english.pdf]

current issues in molecular biology

MDPI

Table S1. Search strategy.

Step	Search history
#1	"Stomatitis"[MeSH Terms] OR ("Stomatitis"[MeSH Terms] OR "Stomatitis"[All Fields] OR "stomatitides"[All Fields] OR ("Stomatitis"[MeSH Terms] OR "Stomatitis"[All Fields] OR ("oral"[All Fields] AND "mucositis"[All Fields]) OR "oral mucositis"[All Fields]) OR ("Stomatitis"[MeSH Terms] OR "Stomatitis"[All Fields] OR ("mucositides"[All Fields] AND "oral"[All Fields])) OR ("Stomatitis"[MeSH Terms] OR "Stomatitis"[All Fields] OR ("oral"[All Fields] AND "mucositides"[All Fields])) OR ("Stomatitis"[MeSH Terms] OR "Stomatitis"[All Fields] OR "oromucositis"[All Fields]) OR ("Stomatitis"[MeSH Terms] OR "Stomatitis"[All Fields]) OR ("Stomatitis"[MeSH Terms] OR "Stomatitis"[All Fields] OR ("mucositis"[All Fields] AND "oral"[All Fields]) OR "mucositis oral"[All Fields]))
#2	("Curcuma"[Mesh]) OR (Curcumas OR Curcuma zedoaria OR Curcuma zedoarias OR Zedoaria, Curcuma OR Zedoary zedoaria OR Zedoary Zedoarias OR Zedoaria, Zedoary OR Curcuma longa OR Curcuma longas OR longa, Curcuma OR Tumeric OR Tumerics OR Turmeric OR Turmerics)
#3	(((((((randomized controlled trial[Publication Type]) OR controlled clinical trial[Publication Type]) OR randomized[Title/Abstract]) OR randomly[Title/Abstract]) OR trial[Title/Abstract]) OR groups[Title/Abstract])
#4	#1 AND #2 AND #3

Table S2. Inclusion and exclusion criteria for the review.

**Inclusion criteria**	**Exclusion criteria**
Radiotherapy (RT)- and/or chemotherapy (CT)-induced oral mucositis on patients with head and neck cancers	Oral mucositis patients not induced by radiotherapy or chemotherapy
Comparison of the effect of curcumin with placebo or other medication	Nonrandomized trials, case reports, reviews, abstract, conference papers, animal or cell experiments, and letters
Randomized clinical trials	

Table S3. Studies excluded after full text assessment with corresponding main reason of exclusion.

Author	Year	Title	Main reason for exclusion
Manjusha et al.[85]	2014	Effectiveness of Indian Turmeric Powder with Honey as Complementary Therapy on Oral Mucositis : A Nursing Perspective among Cancer Patients in Mysore	Not randomized controlled trial
Rita et al. [86]	2022	Comparative randomized trial study about the efficacy of photobiomodulation and curcumin antimicrobial photodynamic therapy as a coadjuvant treatment of oral mucositis in	Curcumin was only used as a photosensitive agent

		oncologic patients: antimicrobial, analgesic, and degree alteration effect	
Pires et al. [87]	2020	Photobiomodulation and photodynamic therapy for the treatment of oral mucositis in patients with cancer	Curcumin was only used as a photosensitive agent
Pinheiro et al. [88]	2019	Photobiomodulation Therapy in Cancer Patients with Mucositis: A Clinical Evaluation	Curcumin was only used as a photosensitive agent
Xavier et al. [89]	2018	Randomized clinical trial of a mucoadhesive formulation containing curcuminoids (Zingiberaceae) and Bidens pilosa Linn (Asteraceae) extract (FITOPROT) for prevention and treatment of oral mucositis - phase I study	Without interested outcome
Sirikorn et al. [90]	2022	Prophylactic management of radiation-induced mucositis using herbal mouthwash in patients with head and neck cancer: an assessor-blinded randomized controlled trial	Not treating with curcumin

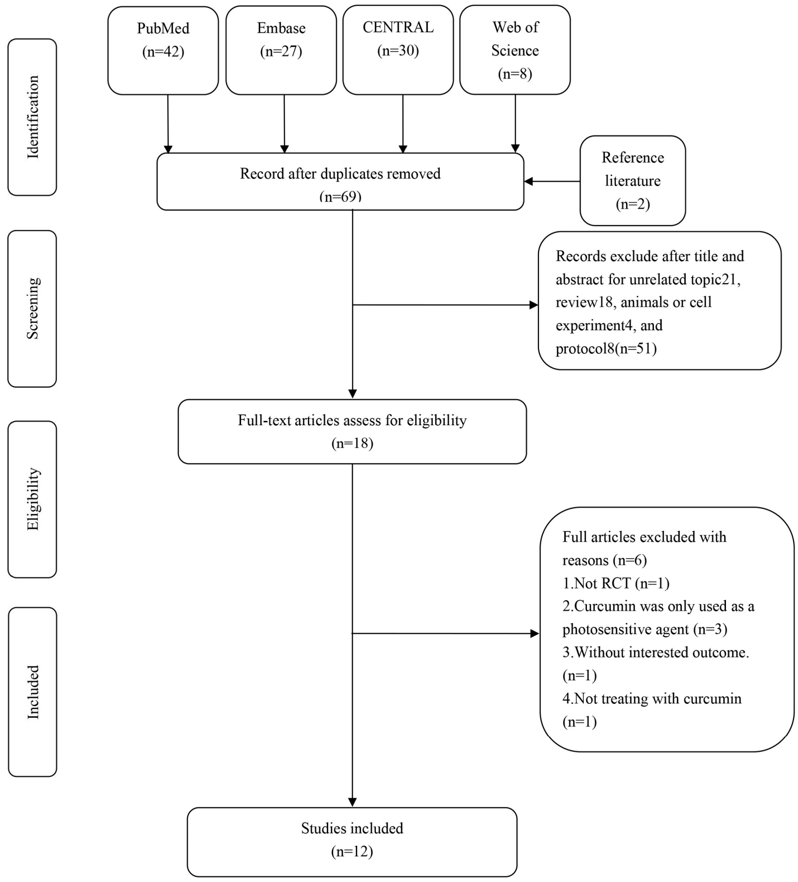

Figure S1. The flowchart of the research selection process.

Table S4. Risk of Bias Assessment for RCTs using the Cochrane Risk of Bias Tool 2.0.

Study	Sureshrao et al.(2014) [43]	Karthikeya Patil et al. (2015) [44]	Mansourian et al. (2015) [45]	Shivayogi Charantimath et al.(2016) [46]	Delavarian et al. (2019) [47]	Arun et al. (2019) [48]	Tej et al. (2021) [37]	Swikant et al. (2021) [38]	Seyed et al. (2021) [40]	Thomas (2022) [39]	Farshid et al. (2023) [42]	Vahid Ramezani et al. (2023) [41]

Randomization Process	Low	Some concerns	Low	Some concerns	Low	Low	Low	Low	Low	Low	Low	Low
Deviations From Intended Interventions	Low	Low	Low	Low	Low	Low	Low	Low	Low	Low	Low	Low
Missing Outcome Data	Low	Low	Low	Low	Low	Low	Low	High	Low	Low	Low	Low
Measurement of the Outcome	Low	High	Some concerns	High	Low	Some concerns	Low	Low	Low	Some concerns	Low	Low
Selection of the Reported Results	Low	Low	Low	Low	Low	Low	Low	Low	Low	Low	Some concerns	Some concerns
Overall	Low	High	Some concerns	High	Low	Some concerns	Low	High	Low	Some concerns	Some concerns	Some concerns
