# Supplementary material for: Effects of Curcumin on Radiation/Chemotherapy-Induced Oral Mucositis: Combined Meta-Analysis, Network Pharmacology, Molecular Docking, and Molecular Dynamics Simulation
Source: Curr Issues Mol Biol. 2024 Sep 20;46(9):10545–69. doi: 10.3390/cimb46090625 (PMC11431004; doi:10.3390/cimb46090625)
Supplement: Supplementary file 1 [file cimb-46-00625-s001.zip › PRISMA_2020_flow_diagram_new_SRs_v1.pdf]

PRISMA 2020 flow diagram for new systematic reviews which included searches of databases and registers only

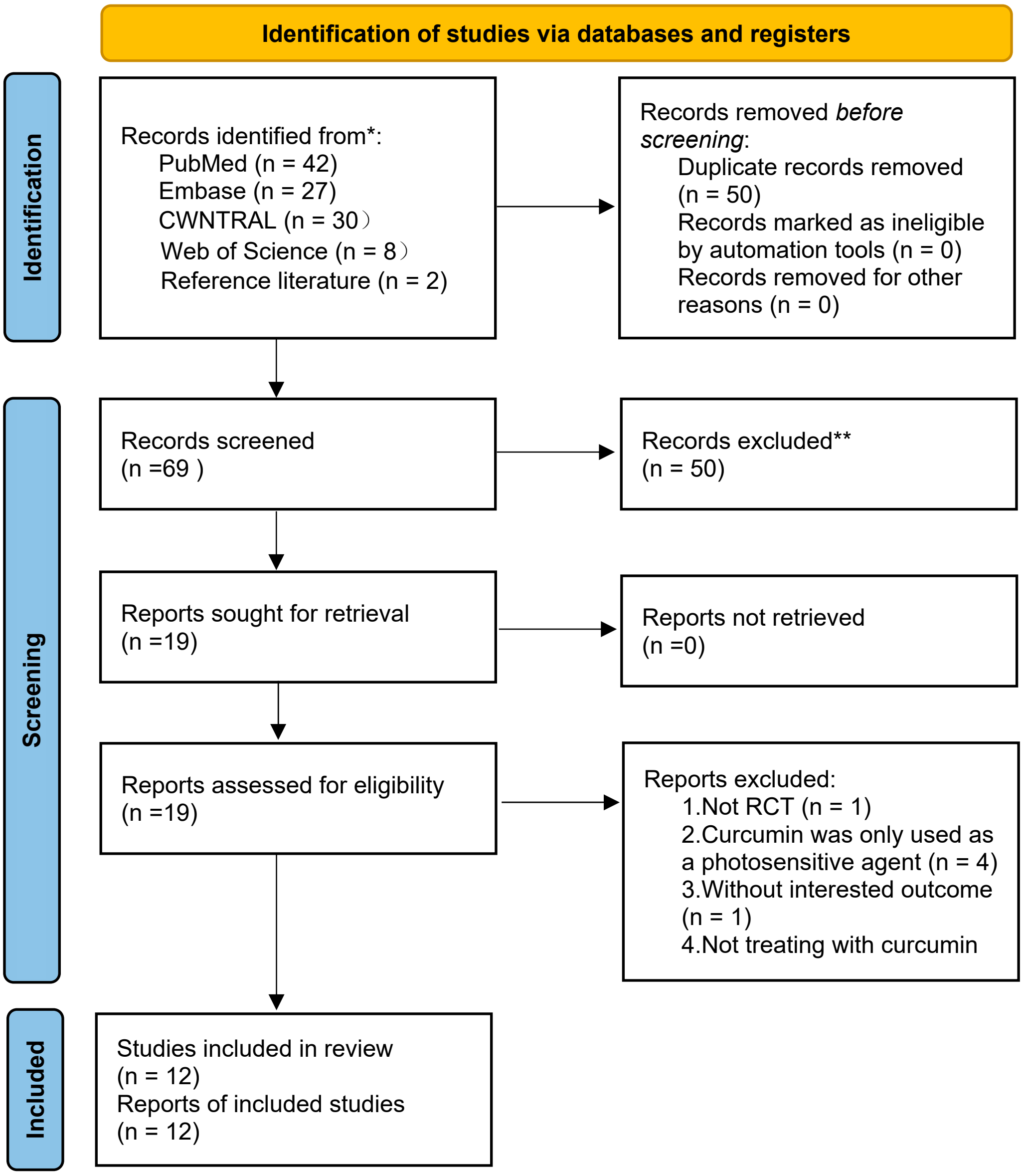

*Consider, if feasible to do so, reporting the number of records identified from each database or register searched (rather than the total number across all databases/registers).

**If automation tools were used, indicate how many records were excluded by a human and how many were excluded by automation tools.

From: Page MJ, McKenzie JE, Bossuyt PM, Boutron I, Hoffmann TC, Mulrow CD, et al. The PRISMA 2020 statement: an updated guideline for reporting systematic reviews. BMJ 2021;372:n71. doi: 10.1136/bmj.n71

For more information, visit: http://www.prisma-statement.org/
